# Associations Between Parental Occupational Balance, Subjective Health, and Clinical Characteristics of VLBW Infants

**DOI:** 10.3389/fped.2022.816221

**Published:** 2022-03-01

**Authors:** Mona Dür, Anna Röschel, Christiane Oberleitner-Leeb, Verena Herrmanns, Elisabeth Pichler-Stachl, Barbara Mattner, Silvia-Desiree Pernter, Martin Wald, Berndt Urlesberger, Herbert Kurz, Thomas Frischer, Karl Zwiauer, Inu Sarah Matter, Angelika Berger

**Affiliations:** ^1^Department of Health Sciences, IMC University of Applied Sciences Krems, Krems, Austria; ^2^Division of Neonatology, Department of Pediatrics and Adolescent Medicine, Pediatric Intensive Care and Neuropediatrics, Comprehensive Center for Pediatrics, Medical University of Vienna, Vienna, Austria; ^3^Duervation, Krems, Austria; ^4^Department of Pediatrics, Clinic Donaustadt, Gesundheitsverbund Vienna, Vienna, Austria; ^5^Department of Pediatrics With Neonatology, St. Josef Hospital Vienna, Vienna, Austria; ^6^Division of Neonatology, Department for Pediatrics and Adolescent Medicine, Medical University of Graz, Graz, Austria; ^7^Division of Neonatology, Department of Pediatrics and Adolescent Medicine, Pediatric Intensive Care, Wilhelminen Hospital, Vienna, Austria; ^8^Division of Neonatology, Department of Pediatrics and Adolescent Medicine, University Hospital Salzburg, Salzburg, Austria; ^9^Faculty for Medicine, Sigmund Freud University, Vienna, Austria; ^10^University Hospital for Pediatrics and Adolescent Medicine, University Hospital St. Pölten, Sankt Pölten, Austria; ^11^Karl Landsteiner University for Health Sciences, Krems, Austria

**Keywords:** preterm infants, neonatal intensive care, mixture of activities, informal caregivers, neonatology and pediatric intensive care

## Abstract

**Objective:**

Very low birthweight (VLBW) infants have an increased risk of mortality and frequently suffer from complications, which affects parental occupational balance. Occupational balance is the satisfaction with one's meaningful activities, which include everyday activities that people need to, want to, and are expected to do. In contrast to work-life balance, the construct of occupational balance addresses different activities equally and it applies to all persons, regardless of whether they are working or not. Parental occupational balance might be related to parents' and VLBW infants' health. Therefore, the objective of this study was to investigate associations between parental occupational balance, subjective health, and clinical characteristics of VLBW infants.

**Methods:**

A cross-sectional multicenter study was conducted in six Austrian neonatal intensive care units. Occupational balance and subjective health of parents of VLBW infants were assessed with six self-reported questionnaires. The following clinical characteristics of VLBW infants were extracted from medical records: gestational age, birthweight, Apgar scores, Clinical Risk for Babies II Score, and complications of prematurity. Spearman's rank coefficients were calculated.

**Results:**

In total, 270 parents, 168 (62%) female and their VLBW infants, 120 (44%) female, were included in this study. Parents' mean age was 33.7 (±6.0) years, mean gestational age of VLBW infants was 27 + 3 (±2) weeks. Associations between parental occupational balance, subjective health, and clinical characteristics of VLBW infants were identified (*r*_s_ = 0.13 – 0.56; *p* ≤ 0.05), such as the correlation between occupational areas (*r*_s_ = 0.22, p ≤ 0.01), occupational characteristics (*r*_s_ = 0.17, *p* = 0.01), and occupational resilience (*r*_s_ = 0.18, *p* ≤ 0.01) with bronchopulmonary dysplasia of VLBW infants.

**Conclusion:**

Occupational balance is associated with parents' and VLBW infants' health. Interventions to strengthen parental occupational balance might increase parental health and thereby also improve health and developmental outcomes of their VLBW infants.

## Introduction

According to the global action report “Born Too Soon,” 15 million infants per year (i.e., 1 in 10) are born preterm (born <37 weeks of gestation) (1). Each year, more than one million children die due to complications of prematurity. Especially very low birthweight (VLBW, <1,500 g) (2) preterm infants have an increased risk of mortality and suffer from various complications and neurodevelopmental disabilities (3–6). The child's entry into life is marked by intense struggle for survival and is accompanied by extraordinary parental stress (7–10). Parents of these children experience high caregiver burden and restricted subjective physical and mental health (11–17). In a vicious circle, the strained parental physical and mental health can adversely affect the children's development (12, 18). Additionally, they have to drastically adapt their everyday lives since their daily routines are predominantly determined by caring activities (19, 20) which restricts their own time for meaningful activities (21–23).

In occupational therapy and occupational science, meaningful activities, also called occupations, refer to everyday activities that people do as individuals, that have meaning and purpose and include activities people need to, want to and are expected to do (24). Deviations in the engagement in meaningful activities cause changes of occupational balance (25). Occupational balance is the satisfaction with the engagement in one's meaningful activities (25, 26). In contrast to work-life balance, the construct of occupational balance addresses different activities equally (27) and it applies to all persons, regardless of whether they are working or not. This becomes even more important as a considerable proportion of parents do not work for a couple of months after their child was born. There is evidence that occupational balance is associated with subjective health and quality of life (26–31). Effects of occupational balance on subjective health and quality of life have recently been identified (26).

Additionally, parental health and well-being are associated with their children's health and well-being (32–34). Occupational balance of caregivers has shown to potentially influence both, their own health and the health of the person they cared for (22, 35). There is increasing evidence, that occupational balance is clinically relevant in pediatrics, especially when it comes to parents of children with complex caring needs or critical health (22, 23, 36–39). Furthermore, a previous study indicated that there might be a link between mothers' occupations and their children's health as well (40).

In summary, parental occupational balance might be associated with children's health and well-being. However, there is limited research in this field. Therefore, the objective of this study was to investigate associations between parental occupational balance, subjective health, and clinical characteristics of VLBW infants.

## Participants and Methods

A cross-sectional multicenter study was conducted to investigate associations between parental occupational balance, subjective health, and clinical characteristics of VLBW infants. This study was part of a larger research project on occupational balance (23) that applied a longitudinal design with measurements at two points in time (1st within 14 days after birth, 2nd within 14 days before discharge). For the current study, data from the first measurement time point was analyzed.

### Study Population

From 2016 to 2018, data was collected in six neonatal intensive care units (NICUs) in Austria. Parents with sufficient German language skills and their preterm infants (born <37 weeks of gestation) with a VLBW (<1,500 g) were included in this study. Parental neuro-motor or psychiatric diseases increased parental psychological burden (based on the subjective evaluation of the responsible pediatrician and the clinical psychologists) and the death of an infant were exclusion criteria.

### Data Collection

Parents and VLBW infants treated in one of the participating centers were screened for eligibility. Eligible parents of VLBW infants were informed about study procedures by pediatricians, occupational therapists [including the first author (MD)], clinical psychologists, speech and music therapists, and nurses of the NICU. Where possible, eligible mothers and fathers were informed personally about the study and invited for participation. If one parent could not be informed personally, they were informed about the study through the other parent and invited to participate.

Subsequently, they were asked to give written and verbal consent, as described elsewhere (41). Participants were asked to complete a sociodemographic data form and to complete a set of self-reported questionnaires to assess occupational balance and subjective health. Eligible parents were asked to participate and to complete the set of questionnaires within the first 2 weeks after birth and within 2 weeks before discharge. Filling in the set of questionnaires took ~ 45 minutes.

### Measures

The main variables of this research were occupational balance, parental subjective health, and clinical characteristics of VLBW infants.

Occupational balance was assessed with the Occupational Balance in Informal Caregivers (OBI-Care) (41) questionnaire. It consists of 22 items, assessing the following dimensions of occupational balance: occupational areas (subscale OBI-Care OA), occupational characteristics (subscale OBI-Care OC), and occupational resilience (subscale OBI-Care OR). All items are scored on a five-choice response scale. Subscales are sum scales of the raw data of scale specific items and range from 5 to 45 (subscale 1), 5 to 35 (subscale 2) and 5 to 30 (subscale 3). Low total scores indicate high satisfaction with one's occupational balance whereas high total scores indicate low satisfaction with one's occupational balance (41).

According to the World Health Organization, “ [...] health is a state of complete physical, mental and social well-being and not merely the absence of disease or infirmity” (42). To cover these various dimensions of health, we measured parental subjective health with several questionnaires. The 12-item version of the Short-Form 36 Health Survey (SF-12) (43) was applied to assess subjective health. Twelve items are used to assess physical health (subscale SF-12 physical health) and mental health (subscale SF-12 mental health). Scoring for each item varies from two- to five-choice response categories. For both subscales, a total score of 100 is the best score and indicates no restrictions due to physical or mental health conditions (43, 44). To assess postnatal depression, the Edinburgh Postnatal Depression Scale (EPDS) (45) was applied. The EPDS is a ten-item questionnaire that covers various clinical symptoms of depression. Achievable scores range from 0 to 30. High total scores indicate more symptoms of depression (45).

Anxiety, stress, and social support were assessed as further measures for mental and social health. The State-Trait Anxiety Inventory (STAI) (46) was used to assess anxiety. The questionnaire consists of 40 items based on a four-point Likert scale and distinguishes between state anxiety (subscale STAI state) and trait anxiety (subscale STAI trait). Scores range from 20 to 80 for each subscale. High total scores for each subscale indicate a heightened feeling of anxiety (46). Stress was assessed with the Parental-Stress-Index (EBI) (47). Forty-eight items, summarized into seven subscales related to parents and five subscales related to children, provide information about perceived stress. Four subscales related to parental bonding, health, isolation, and personal restrictions were applied to this study (subscales EBI bonding, EBI health, EBI isolation, EBI personal restriction). Each subscale ranges from 4 to 20. High total scores indicate a high level of perceived stress (47). The short form of the social support questionnaire (F-SozU) (48) was applied to assess social support. The F-SozU consists of 14 items with five-choice response categories. A low total score indicates low social support (48). For all questionnaires, the German version was used for data collection. Total scores were calculated according to the manual's guidelines.

Additionally, the following clinical characteristics of VLBW infants which are known to predict or influence mortality (49–51) and morbidity (49, 52), were extracted from medical records: sex, birthweight (BW), gestational age (GA), Apgar scores at 5 min (APGAR-5) (53), Clinical Risk for Babies II Score (CRIB II) (54), multiple births, and severe complications of prematurity (8), such bronchopulmonary dysplasia (BPD) (55, 56), defined as oxygen requirement at 36 weeks postmenstrual age; necrotizing enterocolitis (NEC) ≥ Bell's stage 2 (57); severe intraventricular hemorrhage (IVH) defined as grade 3 or 4 according to Volpe (58); retinopathy of prematurity (ROP) defined as stage 3 and above according to the International Classification of Retinopathy of Prematurity revisited (59), requiring intervention; cystic periventricular leukomalacia (PVL) (60); cerebral seizures (CS) (61); and surgical interventions (SI). Severe complications of prematurity were collected at hospital discharge.

### Statistical Analysis

The Statistical Package for Social Sciences (SPSS) version 26.00 (62) was used for statistical analyses. Participants who did not complete the OBI-Care as well as second and third born twins and triplets were excluded from analyses. Data was presented with descriptive statistics, including means and standard deviations (SD) for normally distributed data, medians, and interquartile ranges (IQR) for non-normally distributed data and counts and percentages for dichotomous data. Potential differences of statistical significance between mothers and fathers were identified by conducting Mann–Whitney *U*-tests for independent samples. Hodges–Lehmann estimates were applied to determine the difference. Spearman's rank correlation coefficients (*r*_s_) were calculated to investigate associations between occupational balance (OBI-Care OA, OBI-Care OC, and OBI-Care OR), physical and mental health (SF-12 physical health and SF-12 mental health), postnatal depression (EPDS), anxiety (STAI state and STAI trait), stress (EBI bonding, EBI health, EBI isolation, EBI personal restriction), social support (F-SozU), and clinical characteristics of VLBW infants (GA, APGAR-5, CRIB II, BPD, NEC, IVH, ROP, PVL, CS, SI). Spearman's rank correlation coefficients ≤ 0.30 indicated a weak, 0.31–0.69 a moderate and ≥0.70 a strong association (63). Alpha's level of significance was set at 0.05.

### Ethical Considerations

This study was approved by the following ethics committees: Ethics committee of Medical University of Vienna (1170/2015 Version 2; 1891/2015), City of Vienna (15-255-VK), Lower Austria (GS1-EK-4/461-2017), University of Salzburg (E_2168_4_2017), and Medical University of Graz (29-395 ex 16_17). Participants' written and verbal informed consent was obtained.

## Results

### Participants

**Figure 1:**
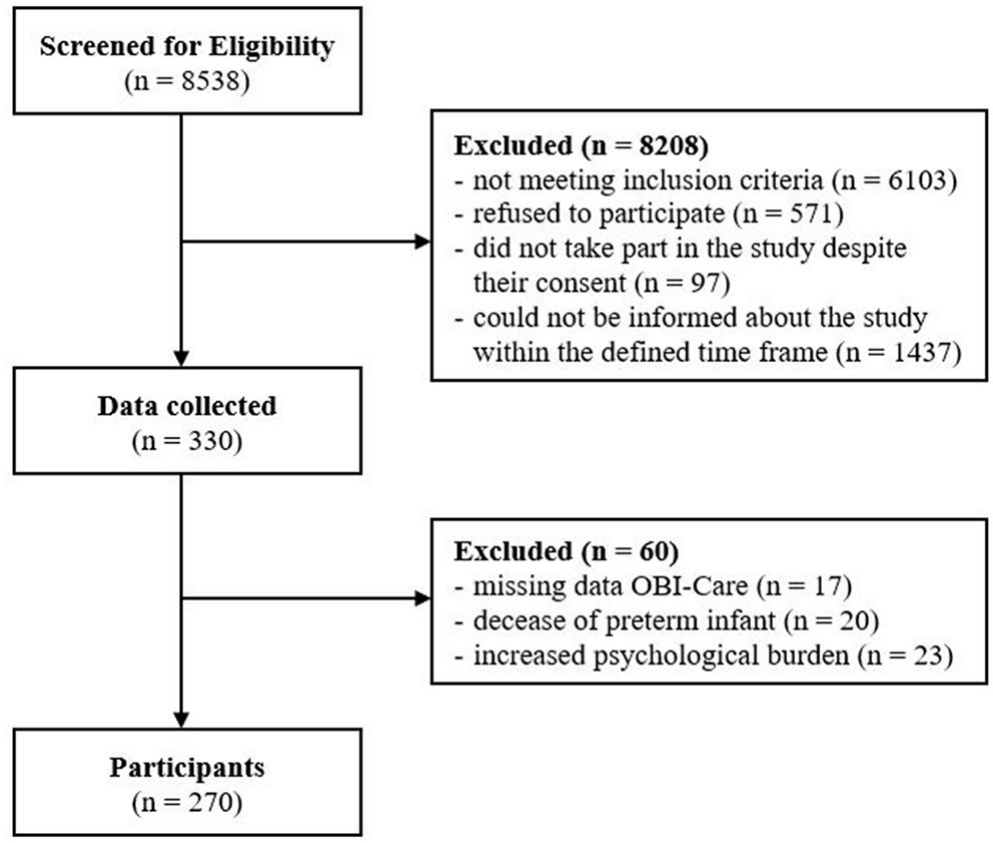
Flow diagram.

A total of 2,435 parents of VLBW infants were eligible for this study. Of those, 330 parents and their first born VLBW infants participated in this study. The data of 60 parents was excluded (Figure 1). Subsequently, data of 270 parents and in total 270 VLBW infants was included for analysis. Two hundred and sixteen (80%) of the included VLBW infants were single births, 52 (19%) were twins and two (1%) were triplets.

Several parents did not take part in the study despite their consent (*n* = 97) since they were not able to fill in the questionnaire within the defined period of 14 days after birth. A large number of parents (*n* = 1,437) could not be informed about the study within the defined period, since they came to the NICU at night, the weekend or very early in the morning due to family, work and other obligations. Based on the personnel resources during off-peak times, there was no staff to inform eligible parents about the study.

Demographic and clinical characteristics of included parents and VLBW infants are presented in Table 1.

**Table 1:** Sample characteristics.

	**Total**	**Female**	**Male**
**Parents**			
Sex, *n* (%)	270 (100)	168 (62)	102 (38)
Mean age (± SD)	33.7 (± 6.0)	33.0 (± 5.7)	35.0 (± 6.4)
Employment status, *n* (%)^a^
Student	5 (2)	3 (2)	2 (2)
Parental leave	156 (58)	153 (91)	3 (3)
Self-employed	26 (7)	7 (4)	19 (19)
Employed	87 (32)	9 (5)	78 (77)
Unemployed	5 (2)	3 (2)	2 (2)
Not specified	5 (2)	1 (1)	4 (4)
**Measures and median scores (IQR)**			
**Occupational balance median (IQR)**			
OBI-Care OA^*^	25 (18.8–31.0)	26 (20.0–31.0)	23 (18.0–29.0)
OBI-Care OC	18 (14.0–22.0)	18 (14.0–22.8)	17.5 (14.0–21.0)
OBI-Care OR	14 (11.0–18.0)	14 (11.0–18.0)	14 (11.0–18.0)
**Subjective health median (IQR)**			
SF12—physical health^*^	40.2 (31.3–53.8)	34.3 (27.0–40.1)	54.8 (51.3–56.8)
SF12—mental health	47.8 (37.6–55.0)	47.3 (36.1–54.1)	49.1 (38.4–55.8)
EPDS^*^	10.0 (5.0–15.0)	11.0 (7.0–16.0)	6.0 (4.0–11.0)
STAI state^*^	43.0 (35.0–50.0)	47.0 (36.0–52.0)	40.0 (32.3–46.8)
STAI trait^*^	37.0 (30.0–44.3)	37.0 (31.0–46.0)	35.0 (30.0–39.0)
EBI bonding	8.0 (6.0–11.0)	8.0 (5.0–11.0)	9.0 (6.0–11.0)
EBI health^*^	10.0 (7.0–13.0)	11.0 (7.0–14.0)	8.0 (5.0–11.0)
EBI isolation	9.0 (7.0–12.0)	9.0 (7.0–12.0)	9.0 (5.5–12.0)
EBI personal restriction	8.0 (5.0–11.0)	8.0 (5.0–11.0)	7.0 (5.0–11.0)
F-SozU	66.0 (60.0–69.0)	67.0 (60.0–69.0)	64.0 (57.0–69.0)
**VLBW infants**			
Sex, *n* (%)	270 (100)	120 (44)	150 (56)
Mean gestational age (± SD)	27+3 (± 2)	27+3 (± 1)	27+3 (± 2)
Mean birthweight in gram (± SD)	1,039.9 (± 286.7)	1,021.0 (± 285.0)	1,054.9 (± 288.1)
Mean APGAR-5 (± SD)^*^	8.5 (± 1.1)	8.3 (± 1.1)	8.6 (± 1.1)
Mean CRIB II (± SD)^*^	8.4 (± 3.7)	7.8 (± 3.5)	8.9 (± 3.8)
Complications of prematurity, *n* (%)^a^	BPD 26 (10)	CS 3 (1)	IVH 39 (14)
	NEC 16 (6)	PVL 4 (1)	ROP 59 (22)
	SI 41 (15)		

a *Multiple answers possible*;

* *significant gender differences; APGAR-5, Apgar score at 5 min; BPD, bronchopulmonary dysplasia; CRIB II, Clinical Risk for Babies II Score; CS, cerebral seizures; IVH, intraventricular hemorrhage; EBI, Parental-Stress-Index; EPDS, Edinburgh Postnatal Depression Scale; F-SozU, Social Support Questionnaire; Max, maximum; Min, minimum; NEC, necrotizing enterocolitis; PVL, periventricular leukomalacia; OBI-Care, Occupational Balance in Informal Caregivers Questionnaire; OA, occupational areas; OC, occupational characteristics; OR, occupational resilience; ROP, retinopathy of prematurity; SD, standard deviation; SF-12, 12 Item Short Form Health Survey 36; SI, surgical interventions; VLBW, very low birthweight*.

Different measures were applied. An overview of applied measures, median scores and interquartile ranges are presented in Table 1.

### Occupational Balance

Included parents of VLBW infants experienced restrictions in their occupational balance. Less than 10% of the parents reached scores indicating a maximum of occupational balance (lowest achievable score) for each subscale. Occupational areas had a median score of 25.0 (IQR = 18.8–31.0) and ranged from 9.0 to 45.0. Occupational characteristics had a median score of 18.0 (IQR = 14.0–22.0) and ranged from 7.0 to 35.0 and occupational resilience had a median score of 14 (IQR = 11.0–18.0) and ranged from 6.0 to 27.0. Significant differences between mothers and fathers were identified in occupational areas (*p* = 0.03). Mothers achieved higher scores than fathers, indicating more restrictions in occupational areas. There were no significant differences between mothers and fathers in occupational characteristics (*p* = 0.25) and occupational resilience (*p* = 0.97).

Medians and IQR for further measures are shown in Table 1.

### Occupational Balance, Physical and Mental Health, and Postnatal Depression

Associations between occupational balance, physical and mental health and postnatal depression were identified (Table 2). Occupational areas (*r*_s_ = −0.19, *p* = 0.02) and occupational characteristics (*r*_s_ = −0.13, *p* = 0.04) showed weak inverse correlations with physical health, indicating that low scores on OBI-Care OA and OBI-Care OC (high satisfaction) relate to high scores on SF-12 physical health (no restrictions). No correlations were identified between occupational resilience and physical health. Moderate associations were found for all dimensions of occupational balance and mental health. Occupational areas (*r*_s_ = −0.45, *p* ≤ 0.01), occupational characteristics (*r*_s_ = −0.46, *p* ≤ 0.01) and occupational resilience (*r*_s_ = −0.44, *p* ≤ 0.01) correlated inversely with mental health. This means that low scores of the OBI-Care (high satisfaction) were associated with high scores of the SF-12 mental health (no restrictions).

**Table 2:** Spearman's rank correlation coefficients occupational balance and subjective health.

**Occupational balance**	**Subjective health**									
	**SF-12**	**SF-12**		**STAI**	**STAI**	**EBI**	**EBI**	**EBI**	**EBI personal**
	**physical**	**mental**	**EPDS**	**state**	**trait**	**bonding**	**health**	**isolation**	**restriction**	**F-SozU**
OBI-Care OA	−0.188*	−0.454*	0.426*	0.515*	0.409*	0.175*	0.554*	0.487*	0.489*	−0.219*
OBI-Care OC	−0.131*	−0.457*	0.412*	0.519*	0.426*	0.192*	0.556*	0.434*	0.473*	−0.282*
OBI-Care OR	−0.063	−0.437*	0.319*	0.435*	0.373*	0.241*	0.477*	0.436*	0.497*	−0.268*

Bold*, correlation is significant (2-tailed); EBI, Parental-Stress-Index; EPDS, Edinburgh Postnatal Depression Scale; F-SozU, Social Support Questionnaire; OBI-Care, Occupational Balance in Informal Caregivers Questionnaire; OA, occupational areas; OC, occupational characteristics; OR, occupational resilience; SF-12, 12 Item Short Form Health Survey 36; STAI, State-Trait Anxiety Inventory.

All subscales of occupational balance correlated with postnatal depression. Moderate correlations were identified between occupational areas (*r*_s_ = 0.43, *p* ≤ 0.01), occupational characteristics (*r*_s_ = 0.41, *p* ≤ 0.01) and occupational resilience (*r*_s_ = 0.32, *p* ≤ 0.01). Low scores of the OBI-Care were related to low scores of the EPDS (both the favorable scores).

### Occupational Balance, Anxiety, Stress, and Social Support

Occupational balance was found to correlate with anxiety, stress, and social support (Table 2). All dimensions of occupational balance and both dimensions of anxiety correlated moderately with each other. Occupational areas (*r*_s_ = 0.52, *p* ≤ 0.01), occupational characteristics (*r*_s_ = 0.52, *p* ≤ 0.01), and occupational resilience (*r*_s_ = 0.44, *p* ≤ 0.01) correlated with state anxiety and with trait anxiety (*r*_s_ = 0.41, *p* ≤ 0.01; *r*_s_ = 0.43, *p* ≤ 0.01; *r*_s_ = 0.37, *p* ≤ 0.01). This indicates that low scores of the OBI-Care (high satisfaction) were associated with low scores of the STAI (low feeling of anxiety).

Moreover, all subscales of occupational balance were associated with stress. Occupational areas (*r*_s_ = 0.18, *p* ≤ 0.01), occupational characteristics (*r*_s_ = 0.19, *p* ≤ 0.01), and occupational resilience (*r*_s_ = 0.24, *p* ≤ 0.01) correlated weakly with bonding, moderately with health (*r*_s_ = 0.55, *p* ≤ 0.01; *r*_s_ = 0.56, *p* ≤ 0.01; *r*_s_ = 0.48, *p* ≤ 0.01), with isolation (*r*_s_ = 0.49, *p* ≤ 0.01; *r*_s_ = 0.43, *p* ≤ 0.01; *r*_s_ = 0.44, *p* ≤ 0.01) and with personal restriction (*r*_s_ = 0.49, *p* ≤ 0.01; *r*_s_ = 0.47, *p* ≤ 0.01; *r*_s_ = 0.50, *p* ≤ 0.01). Low scores of the OBI-Care were related to low scores of the EBI (both the favorable scores).

Additionally, all subscales of occupational balance correlated with social support. Occupational areas (*r*_s_ = −0.22, *p* ≤ 0.01), occupational characteristics (*r*_s_ = −0.28, *p* ≤ 0.01) and occupational resilience (*r*_s_ = −0.27, *p* ≤ 0.01) showed weak inverse correlations with social support, indicating that low scores of the OBI-Care (high satisfaction) are associated with high scores of social support (high social support).

### Occupational Balance and Clinical Characteristics of VLBW Infants

Occupational balance was found to be associated with one of the selected clinical characteristics of VLBW infants (Table 3). Occupational areas (*r*_s_ = 0.22, *p* ≤ 0.01), occupational characteristics (*r*_s_ = 0.17, *p* = 0.01), and occupational resilience (*r*_s_ = 0.18, *p* ≤ 0.01) correlated weakly with BPD. High scores of the OBI-Care were related to the occurrence of BPD. There was no evidence of associations between occupational balance and the other selected clinical characteristics of VLBW infants, such as IVH or ROP.

**Table 3:** Spearman's rank correlation coefficients occupational balance and clinical characteristics of VLBW infants.

**Occupational balance**	**Clinical characteristics of VLBW infants**											
	**APGAR 5**	**BPD**	**BW**	**CRIB II**	**CS**	**GA**	**IVH**	**MB**	**NEC**	**PVL**	**ROP**	**SI**
OBI-Care OA	0.113	0.217*	−0.042	0.022	−0.009	−0.042	0.050	−0.029	0.011	−0.019	0.012	0.091
OBI-Care OC	0.096	0.165*	−0.046	−0.006	0.011	−0.038	0.098	−0.018	0.000	−0.085	−0.013	0.088
OBI-Care OR	0.087	0.179*	0.017	−0.028	0.022	−0.005	0.014	−0.045	−0.055	−0.043	0.044	0.097

Bold*, correlation is significant (2-tailed); APGAR-5, Apgar score at 5 minutes; BPD, bronchopulmonary dysplasia; BW, birthweight; CRIB II, Clinical Risk for Babies II Score; CS, cerebral seizures; GA, gestational age (WHO Classification); IVH, intraventricular hemorrhage; MB, multiple births; NEC, necrotizing enterocolitis; OBI-Care, Occupational Balance in Informal Caregivers Questionnaire; OA, occupational areas; OC, occupational characteristics; OR, occupational resilience; PVL, periventricular leukomalacia; ROP, retinopathy of prematurity; SD, Standard deviation; SI, surgical interventions; VLBW, very low birthweight.

## Discussion

Research on parental occupational balance is scarce. Two studies on occupational balance were based on the use of qualitative research methods (22, 64). Studies that reported the use of measures of occupational balance in parents included parents of children aged 8 years or younger (36, 37), or did not declare children's age (65). It is unclear whether parents of newborns or children under the age of 1 year were included since this was not described explicitly. To our knowledge this study is the first study that assessed occupational balance in parents of VLBW infants. Within this study we could demonstrate that occupational balance of parents' of VLBW infants is associated with physical and mental health, postnatal depression, and BPD of VLBW infants.

Data from both mothers and fathers indicated restricted occupational balance. Contrary to other studies on occupational balance (66, 67), we could identify significant gender differences for one subscale exclusively. Previous research on parents' emotional response to preterm birth showed that fathers experienced a lower level of distress (7, 34). Nevertheless, it is possible that fathers and mothers of VLBW infants are equally involved in and affected by the care for their VLBW infants. This assumption is consistent with another study that reports comparable psychological burdens for fathers and mothers (12).

In line with previous studies on occupational balance, we found associations between occupational balance and subjective health. However, previous studies on occupational balance included patients with chronic autoimmune diseases (27, 28), mental health problems (29, 31), adults without any health issues (26, 30, 31), and persons aged 55 years or older (26). These studies showed associations between occupational balance and physical, mental, and general health (26–29). To our knowledge, the current study is the first that included parents of VLBW infants. In the current study, occupational balance was found to be associated with postnatal depression, anxiety, and stress. Previous studies also described associations between occupational balance, depression, anxiety, and stress in patients with mental health problems (68) and persons aged 55 (26) or older. Additionally, occupational balance was associated with social support.

Initial evidence for associations between parental occupational balance and clinical characteristics of VLBW infants was found. Occupational balance and the occurrence of BPD correlated significantly. Previous studies also reported associations with infants' pulmonary diseases and more symptoms of depression in mothers of VLBW infants (69, 70). VLBW infants with BPD have an increased risk of mortality. Additionally, BPD jeopardizes pulmonary and neurosensory development and long-term outcomes of children and is often the consequence of multiple treatments such as prolonged ventilatory support (71–74). Due to these multiple complications and treatments, parents of VLBW infants with BPD might experience more limitations in the interaction with their VLBW infant and their everyday lives compared to parents of VLBW infants with other complications of prematurity. This provides a possible explanation why BPD was exclusively associated with parental occupational balance in this study. However, further research is needed to explore potential reasons for an association between parental occupational balance and BPD.

Further clinical characteristics of VLBW infants (APGAR, CRIB II, GA, NEC, IVH, ROP, PVL, CS, and SI) did not correlate with parental occupational balance. However, these are important clinical characteristics related to prematurity (8, 75, 76) that were found to affect parents of VLBW infants. For example, mothers of infants diagnosed with ROP were described to have higher levels of postnatal depression and anxiety (77). Parental health and clinical characteristics of VLBW infants were found to be associated in previous research (32, 78–80). Further studies provided evidence that children of mentally ill parents run a significantly greater risk of developing poor mental and physical clinical characteristics (33, 34). Furthermore, there might be an association between parental meaningful activities and parental and infant health (40).

Regarding these findings, a better understanding of occupational balance in parents of VLBW infants might be beneficial for health professionals working in NICUs. Parents at NICUs are encouraged to engage in caring activities such as feeding, kangarooing and skin-to-skin care (81–83). Nevertheless, health professionals working in NICUs do not usually carry out treatment to strengthen parents' occupational balance. Occupational therapists are experts in occupational balance and refer to occupational balance and meaningful activities as outcome, as well as intervention. We therefore recommend that occupational therapists (27, 84) and other health professionals who work at NICUs assist parents of VLBW infants in engaging in meaningful activities and set further interventions to strengthen their occupational balance. Interventions to increase occupational balance of parents of VLBW infants might strengthen parental subjective health and thereby also improve health and developmental outcomes of their VLBW infants.

This study has strengths and limitations. The multicenter design yielded a high diversity within the sample and a higher sample size than expected, which strengthens the generalizability of the results. Correlation analyses were conducted exclusively to identify associations between parental occupational balance and the following measures: physical and mental health, postnatal depression, anxiety, stress, social support, and clinical characteristics of VLBW infants. Due to this explorative approach, we did not adjust for multiple testing. Thus, the results of this study have an explorative character as well. Related studies are warranted to further define the direction and effect size of identified associations (85, 86). Additionally, an inclusion of all twins and triplets into correlation analysis could have led to different results. However, a larger sample size would have increased the probability to find statistically significant associations (87) between parental occupational balance and clinical characteristics of preterm infants. To our knowledge, there are no other studies reporting on measures of occupational balance of parents of full or pre-termed infants which could be used for comparison and to fully understand the effect of having a VLBW infant. Further research is needed to evaluate parental occupational balance and to identify aberrations with clinical relevance.

## Conclusion

Parents of VLBW infants reported restrictions in occupational balance. Associations between parental occupational balance and subjective health, and BPD of VLBW infants were identified. Interventions to strengthen parental occupational balance might have a positive impact on the parent' own and their VLBW infant's health.

## Data Availability Statement

The raw data supporting the conclusions of this article will be made available by the authors, without undue reservation.

## Ethics Statement

This study was approved by the Ethics Committee of Medical University of Vienna (1170/2015 Version 2; 1891/2015), City of Vienna (15-255-VK), Lower Austria (GS1-EK-4/461-2017), University of Salzburg (E_2168_4_2017), and Medical University of Graz (29-395 ex 16_17). Participants' written and verbal informed consent was obtained. Written informed consent to participate in this study was provided by the participants' legal guardian/next of kin.

## Author Contributions

MD conceptualized and designed the study, collected data, coordinated, supervised data collection, carried out the initial analyses, and drafted the initial manuscript and revised the manuscript. AR and IM carried out the initial analyses, discussed the results, drafted the initial manuscript, and revised the manuscript. CO-L collected data, coordinated data collection, supported analysis, and critically reviewed the manuscript. VH, EP-S, BM, and S-DP collected data, coordinated data collection, and critically reviewed the manuscript. MW, BU, HK, TF, and KZ coordinated and supervised data collection, discussed the results, and critically reviewed the manuscript for important intellectual content. AB conceptualized and designed the study, coordinated data collection, and critically reviewed the manuscript for important intellectual content. All authors approved the final manuscript as submitted and agree to be accountable for all aspects of the work.

## Funding

This study was partly funded by the Common Health Targets fund of Rahmen-Pharmavertrag (MD), Ergotherapie Austria (MD), and Verein Unser Kind (MD). The funders had no role in the study design, the collection, analysis, and interpretation of data, the writing of the report, and the decision to submit the article for publication.

## Conflict of Interest

MD was the CEO of company Duervation GmbH. The salary of MD and CO-L were partly covered by the project costs. The remaining authors declare that the research was conducted in the absence of any commercial or financial relationships that could be construed as a potential conflict of interest.

## Publisher's Note

All claims expressed in this article are solely those of the authors and do not necessarily represent those of their affiliated organizations, or those of the publisher, the editors and the reviewers. Any product that may be evaluated in this article, or claim that may be made by its manufacturer, is not guaranteed or endorsed by the publisher.
